# A Chemoenzymatic Approach To Produce a Cyclic Analogue of the Analgesic Drug MVIIA (Ziconotide)

**DOI:** 10.1002/anie.202302812

**Published:** 2023-05-31

**Authors:** Yan Zhou, Peta J. Harvey, Johannes Koehbach, Lai Yue Chan, Alun Jones, Åsa Andersson, Irina Vetter, Thomas Durek, David J. Craik

**Affiliations:** ^1^ ARC Centre of Excellence for Innovations in Peptide and Protein Science Institute for Molecular Bioscience The University of Queensland Brisbane QLD 4072 Australia; ^2^ Institute for Molecular Bioscience The University of Queensland Brisbane QLD 4072 Australia; ^3^ School of Pharmacy Institute for Molecular Bioscience The University of Queensland Brisbane QLD 4072 Australia

**Keywords:** Chemoenzymatic Synthesis, Cyclization, Disulfide Bonds, Enzymes, Peptides

## Abstract

Ziconotide (ω‐conotoxin MVIIA) is an approved analgesic for the treatment of chronic pain. However, the need for intrathecal administration and adverse effects have limited its widespread application. Backbone cyclization is one way to improve the pharmaceutical properties of conopeptides, but so far chemical synthesis alone has been unable to produce correctly folded and backbone cyclic analogues of MVIIA. In this study, an asparaginyl endopeptidase (AEP)‐mediated cyclization was used to generate backbone cyclic analogues of MVIIA for the first time. Cyclization using six‐ to nine‐residue linkers did not perturb the overall structure of MVIIA, and the cyclic analogues of MVIIA showed inhibition of voltage‐gated calcium channels (Ca_V_2.2) and substantially improved stability in human serum and stimulated intestinal fluid. Our study reveals that AEP transpeptidases are capable of cyclizing structurally complex peptides that chemical synthesis cannot achieve and paves the way for further improving the therapeutic value of conotoxins.

## Graphical abstract

A chemoenzymatic method has been developed to prepare backbone cyclic analogues of the ω‐conotoxin MVIIA. The most potent cyclic analogue, cM‐7, shows highly improved stability and maintains the activity of the native peptide.

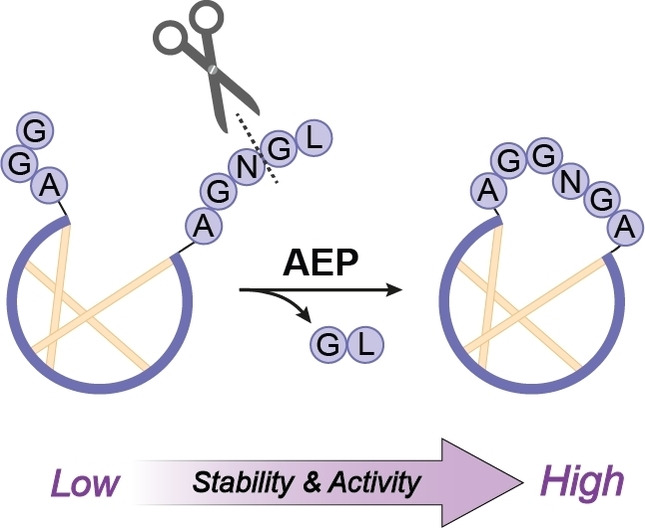

Conotoxins are disulfide‐rich peptides isolated from the venom of marine snails of the *Conus* genus. These peptides have evolved as defence against predators or for paralyzing prey.[^1^, ^2^] Conotoxins range in size from 12 to 60 amino acids and have attracted the interests of pharmaceutical scientists due to their high resistance to proteases and extraordinary selectivity and potency for specific pharmacological targets of therapeutic value.^[1]^ Their high specificity has made them attractive molecular probes during the process of target validation and drug development of bioactive peptides.[^1^, ^2^] Several conotoxins have entered clinical trials as treatments for various neuropathic diseases.^[1]^ The most successful example is ω‐conotoxin MVIIA (ziconotide) which was approved by the US Food and Drug Administration (FDA) in 2004 for the treatment of severe chronic pain.^[3]^

MVIIA, from the venom of the cone snail *Conus magus*, is a highly selective voltage‐gated calcium channel (Ca_V_2.2) inhibitor that controls neurotransmission at numerous synapses.^[4]^ The primary structure of MVIIA comprises 25 residues including six cysteines that form three structure‐stabilizing disulfide bonds (Cys^I^–Cys^IV^, Cys^II^–Cys^V^, Cys^III^–Cys^VI^).^[5]^ Tyr13 and Arg10 in loop 2 are the primary determinants for binding to Ca_V_2.2. Other residues important for the selectivity and activity against the Ca_V_2.2 channel include Lys2, Leu11 and Arg21.[^4b^, ^6^] A recent study suggested that the side effects of MVIIA may result from Met12 in loop 2.^[7]^ The 3D structure of MVIIA, determined by nuclear magnetic resonance (NMR) spectroscopy, shows a triple‐stranded β‐sheet that together with the three disulfide bonds forms a characteristic inhibitor cystine knot (ICK) motif. A β‐bridge formed by residues 1–2 and 14–16 is stabilized by a salt‐bridge between the side chain of Lys2 and Asp14. The intervening regions between the β‐sheet and β‐bridge comprise β‐turns, providing a highly rigid backbone structure that optimally positions the critical side chains (Scheme 1A; PDB code 1mvi).[^6a^, ^8^]

**Scheme 1:**
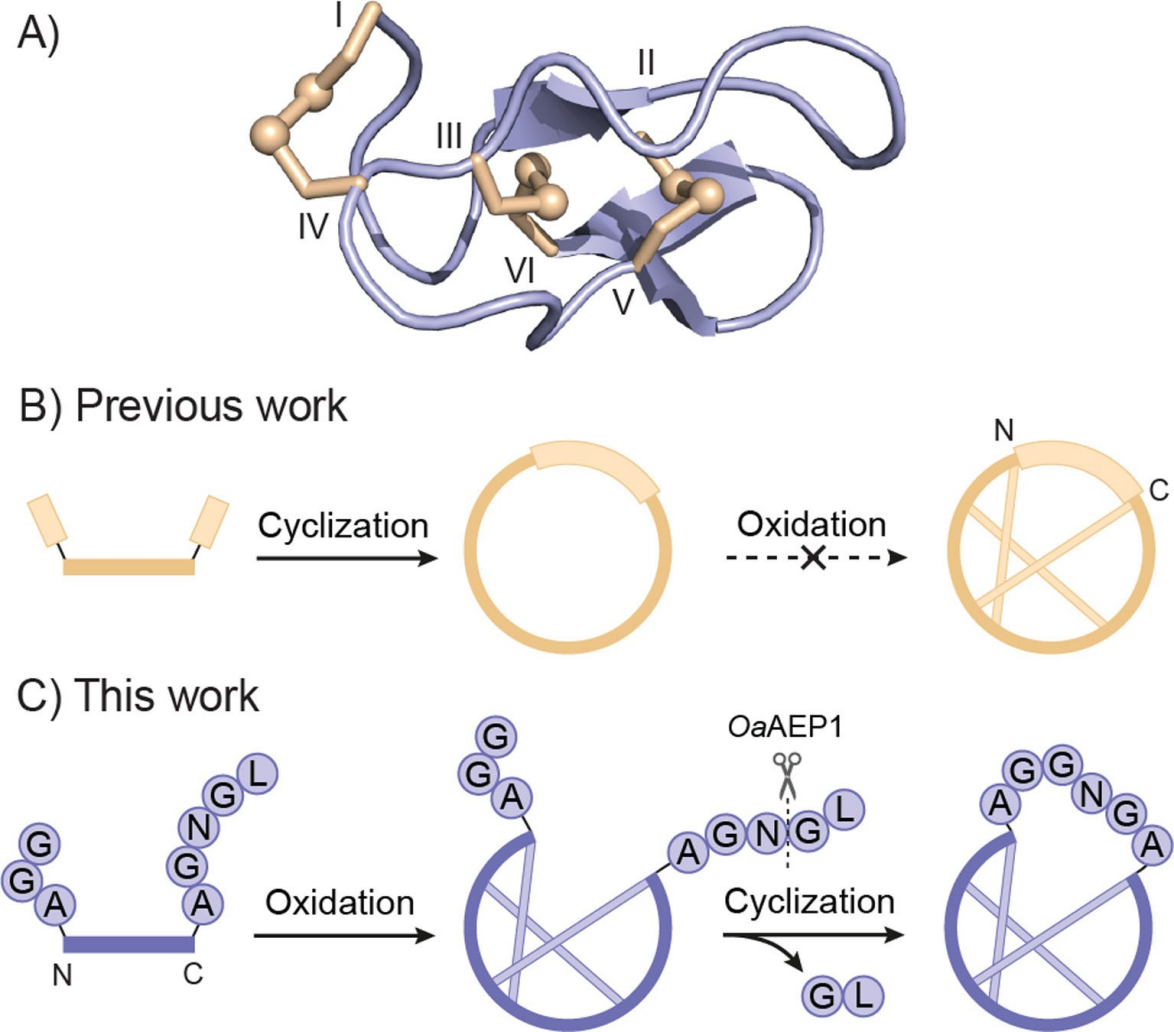
The strategy for making cyclic MVIIA. A) The 3D structure of MVIIA. B) Chemical cyclization prior to oxidation. C) AEP‐mediated cyclization following oxidation.

Cyclization has emerged as a strategy in the pharmaceutical industry to enhance the biopharmaceutical properties of peptides.^[9]^ Compared to linear forms, cyclic peptides are generally more stable because their head‐to‐tail cyclized backbone can engender them with resistance to adverse physiological conditions and prevent degradation by diverse proteases.^[10]^ The traditional strategy to produce cyclic peptides is assembly of a linear precursor by solid‐phase peptide synthesis (SPPS).^[11]^ After cleavage from the solid support, the peptide is then backbone cyclized using amide‐forming chemistries (Scheme 1B), for example using native chemical ligation (NCL). Because most cyclization chemistries are incompatible with unprotected peptides or disulfide bonds (e.g. NCL), the formation of the disulfide bonds to obtain the properly folded product is generally carried out after the backbone cyclization. To date, seven α‐conotoxins have been chemically cyclized and all displayed increased proteolytic stability and some improved pharmacological properties.^[12]^ However, attempts to synthesize a cyclic form of the ω‐conotoxin MVIIA have been challenging—backbone cyclic analogues with a reduced number of disulfide bonds have been reported,^[13]^ but these are expected to be of limited value as disulfide deletion disrupts the integrity of the ICK structure. Additionally, MVIIA has been cyclized through NCL using a four‐residue GGPG linker,^[14]^ but the oxidized product was not structurally validated, nor was its biological activity determined.

Our earlier attempts to produce backbone cyclic MVIIA identified the following synthetic challenges. Firstly, the N‐ and C‐termini of MVIIA are relatively far apart (ca. 10 Å) and are pointing in opposite directions with little flexibility due to the rigidity imposed by the ICK core. This arrangement necessitates an appropriate linker sequence to bridge the gap. Secondly, backbone cyclic analogues of MVIIA with a variety of different linkers do not produce correctly ICK folded molecules under various conditions (as judged by NMR analysis of the reaction products). Although disulfide formation strategies using orthogonal protecting group schemes^[15]^ or diselenide surrogates^[16]^ could be used to direct formation of the ICK structure, we here sought a way to reverse the order of synthetic steps, i.e. to form the disulfides first on a linear precursor peptide, followed by the backbone cyclization step under conditions that do not disrupt the integrity of the disulfide bonds (Scheme 1C).

In this study, an asparaginyl endopeptidase (AEP) was employed to mediate the backbone cyclization of peptide precursors of MVIIA. AEPs cleave C‐terminally at asparagine or aspartic acid residues, after recognition of a compatible Asx‐Gly‐Leu (P1−P1′−P2′) motif.^[17]^ To explore the effect of various linker lengths on cyclization efficiency, structure and activity, five MVIIA analogues were designed that incorporated a linker segment containing five to nine amino acid residues between the N‐ and C‐termini, and were synthesized by SPPS (Scheme 2).

**Scheme 2:**
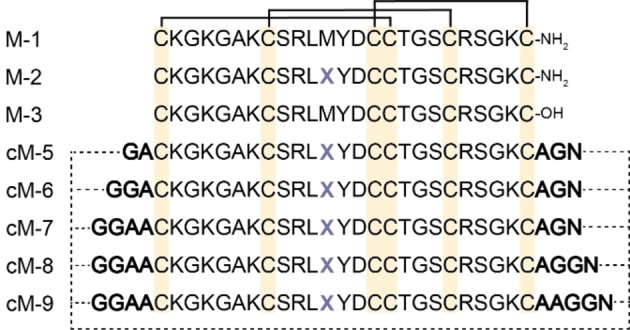
Sequences of designed MVIIA analogues. Disulfide bonds are shown as brackets above the sequences. Five cyclic MVIIA precursors with five‐residue (GAAGN, cM‐5), six‐residue (GGAAGN, cM‐6), seven‐residue (GGAAAGN, cM‐7), eight‐residue (GGAAAGGN, cM‐8) and nine‐residue (GGAAAAGGN, cM‐9) linkers were designed. The dashed line joining the N and C termini together indicates backbone cyclization. The letter X represents norleucine.

Gly and Ala were used primarily as linker residues because they are sterically less demanding and can accommodate a variety of secondary structures. Additionally, Met12 was substituted by the isosteric norleucine to avoid potential problems associated with methionine oxidation that will negatively affect MVIIA activity.^[18]^

The linear control peptides (M1–M3) and precursor peptides (M5–M9) were synthesized by 9‐fluorenylmethyloxycarbonyl‐based SPPS (Figure S1). The peptides were cleaved from the resin and side chain deprotected and purified by HPLC (Figure 1A). The purified peptides were subjected for 48 hours to conditions (0.33 M NH_4_OAc/0.5 M GnHCl, pH 6.5) that facilitate disulfide bond formation, illustrated for M‐7 in Figure 1B, followed by RP‐HPLC purification to obtain the pure folded precursor peptides (Figure S2). The correct isomer was the dominant product which gave a high yield of approximately 25 % after purification. ESI‐MS was used to confirm the oxidation of all cysteines (Figure S3) and 1D ^1^H NMR spectroscopy was used to evaluate all analogues. Analysis of the NMR data showed well‐dispersed resonances in the amide/fingerprint region indicating that all analogues retained the native disulfide bonded connectivity of MVIIA (Figure S7).

**Figure 1:**
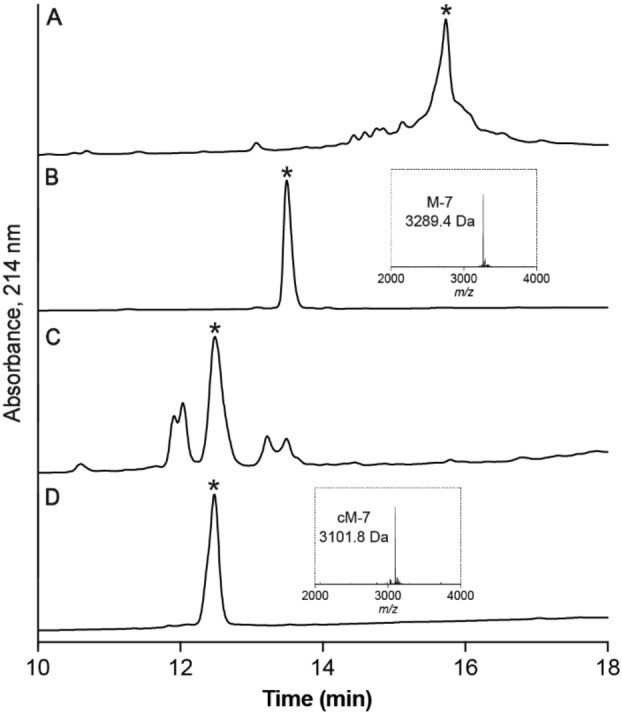
Cyclization/oxidation analytical liquid chromatography profiles. A) The reduced M‐7 after deprotection and RP‐HPLC purification. B) The folded M‐7 (calculated [*M*+H]^+^=3289.4 Da; observed [*M*+H]^+^=3289.4 Da). C) AEP‐medicated cyclization of M‐7. D) The purified cM‐7 (calculated [*M*+H]^+^=3101.4 Da; observed [*M*+H]^+^=3101.8 Da).

The properly folded cyclic MVIIA precursors (100 μM) were incubated with the cyclization‐efficient AEP [C247A]*Oa*AEP1_b_ (1 μM) in sodium phosphate buffer at pH 6.5 for 24 h (Figure 1C), followed by HPLC purification of the cyclic products for further structural characterization and assays (Figure 1D). LC–MS/MS showed all these cyclic MVIIA precursors could be cyclized. Notably, M‐6 and M‐7 with a six or seven amino acid linker had very high cyclization efficiency and the yield of the cyclic peptides after purification was approximately 10 %. However, analogues with eight (M‐8) or nine (M‐9) residues could not be efficiently cyclized and produced substantial amounts of AEP‐hydrolyzed side‐products, indicating that the length of the linker can shift the enzyme activity preference, i.e., hydrolysis vs transpeptidation (Figure S4). Cyclization of M‐5 was extremely slow with little hydrolysis side product and the unreacted linear peptide precursor being the major component after 24 h (Figure S5), Overall, these data highlight that the linker length has a tremendous impact on AEP‐catalyzed cyclization efficiency.

We used peptide mass fingerprinting to further demonstrate that the mass shift seen upon cyclization was indeed the result of backbone cyclization and not an unrelated dehydration event: After reduction and alkylation and trypsin‐digestion, a peptide fragment that covered the cyclized region was found by LC–MS/MS and numerous b‐ and y‐ions corresponding to the peptide were identified based on MS/MS fragmentation pattern confirming cyclization (Figure S6).

Structural comparisons of the native (linear) MVIIA and the cyclic analogues were undertaken by NMR spectroscopy. All the cyclic analogues except cM‐5 displayed sharp, well‐dispersed peaks in the 1D ^1^H NMR spectrum, suggesting they maintained a well‐defined fold after cyclization (Figure S8). The linear M‐5 precursor also showed well‐dispersed spectra, but upon AEP cyclization the little cyclic product that was formed showed broad peaks and poor dispersion in the fingerprint region, suggesting that correct disulfide connectivity had been lost. This observation suggests that the linker of the correctly folded M‐5 is too short, making it a poor substrate for AEP. In this case and over long reaction times, a small fraction of peptide has its disulfides scrambled, resulting in a better AEP substrate and production of scrambled cyclic product cM‐5. Thus, while we anticipate this approach to be generally applicable, highly constrained peptides such as MVIIA may require careful optimization of the cyclization linker length and structure.

cM‐7 showed the highest quality NMR spectra and was also isolated in sufficient quantities to allow further 2D NMR spectroscopy, including 2D TOCSY, NOESY, COSY and HSQC spectra. Full assignment of proton resonances was possible in addition to backbone carbons and amide nitrogens (apart from C5 and D18 due to overlap). Comparison with literature data[^8^, ^19^] showed a remarkable similarity between native MVIIA and the core region of cM‐7 (particularly residues C5–C29, Table S2). Comparison of secondary αH shifts (Figure 2) reveals negligible differences suggesting that the backbone conformation of cM‐7 is identical to that of the native linear peptide. Variable temperature, along with D_2_O exchange, experiments were acquired to indicate residues involved in hydrogen bonding. The temperature coefficients and slowly exchanging amides suggest similar hydrogen bond donor/acceptor pairs as reported in the literature[^8^, ^19^, ^20^] (Table S3) that establish the three short and distorted antiparallel β strands of MVIIA.

**Figure 2:**
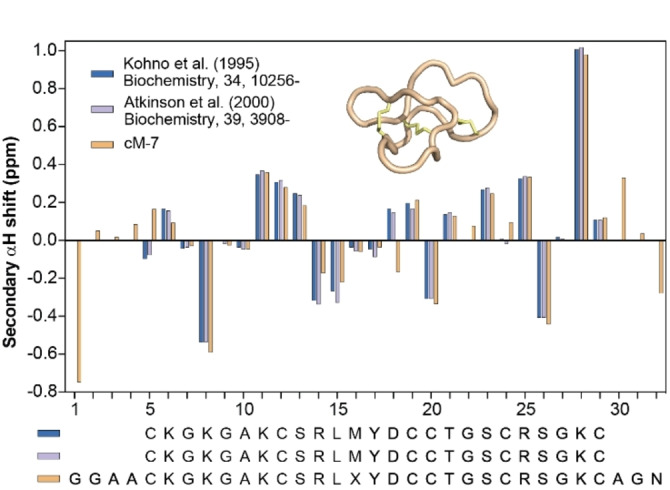
NMR analysis of cM‐7 and comparison to literature values.[^8^, ^19^] Secondary αH chemical shifts are the difference between the measured αH shift and a random coil value for the same residue type.^[21]^ X represents norleucine, for which we used the random coil value of leucine.

To further examine our hypothesis that the order of disulfide formation and backbone cyclization is critical, we prepared a fully reduced cyclic peptide of cM‐7. A series of folding conditions were tested to oxidize this peptide and the reactions were analyzed by LC–MS after 2 days. However, none of the folding products co‐eluted with authentic enzyme‐cyclized cM‐7 (Figure S9), suggesting that they are not correctly folded and that cyclization prior to disulfide formation negatively impacts the ability of the peptide to acquire its ICK structure. This observation contrasts with many other examples of linear and cyclic peptide folding attempts, where usually the cyclic structure folds more efficiently than the corresponding linear counterpart. This is believed to be due to the reduced conformational freedom of the constrained cyclic peptides limiting the number of disulfide configurations, providing a more direct path to the correct pairing.^[22]^

Consistent with previous studies,^[6a]^ the native MVIIA (M‐1) potently inhibited Ca_V_2.2 with an IC_50_ of 0.8±2.5 nM (*n*=7). Interestingly, linear M‐2 (IC_50_=0.1±1.0 nM; *n*=7) showed nearly 10‐fold higher inhibition after methionine was replaced by norleucine. This finding is in agreement with earlier studies and not surprising given that Met12 is next to the critically important Tyr13 and known to be prone to oxidation.^[18]^ Importantly, although we chose to replace methionine in our MVIIA analogues for pharmacological reasons, our AEP‐mediated method is fully compatible with this sensitive amino acid, as is demonstrated by successful cyclization of a Met containing analogue of cM‐7 (Figure S11). Linear M‐3 with a free C‐terminal carboxy group instead of the native carboxamide was slightly less active (IC_50_=1.7±1.2 nM; *n*=7) indicating that modification near the C‐terminus can affect the peptide potency (Figure 3). The cyclic analogue cM‐6 (IC_50_=43.2±1.4 nM; *n*=7) was less active than the native MVllA, but increasing the linker by one residue (cM‐7) could rescue the action on Ca_V_2.2 channel (IC_50_=13.5±1.2 nM; *n*=7). The cyclic analogue cM‐8 was slightly less active than cM‐7 (IC_50_=21.8±1.2 nM; *n*=7), whereas cM‐9 failed to inhibit Ca_V_2.2 channel at the similar concentration (IC_50_=306.6±1.5 nM; *n*=7; Table S4).

**Figure 3:**
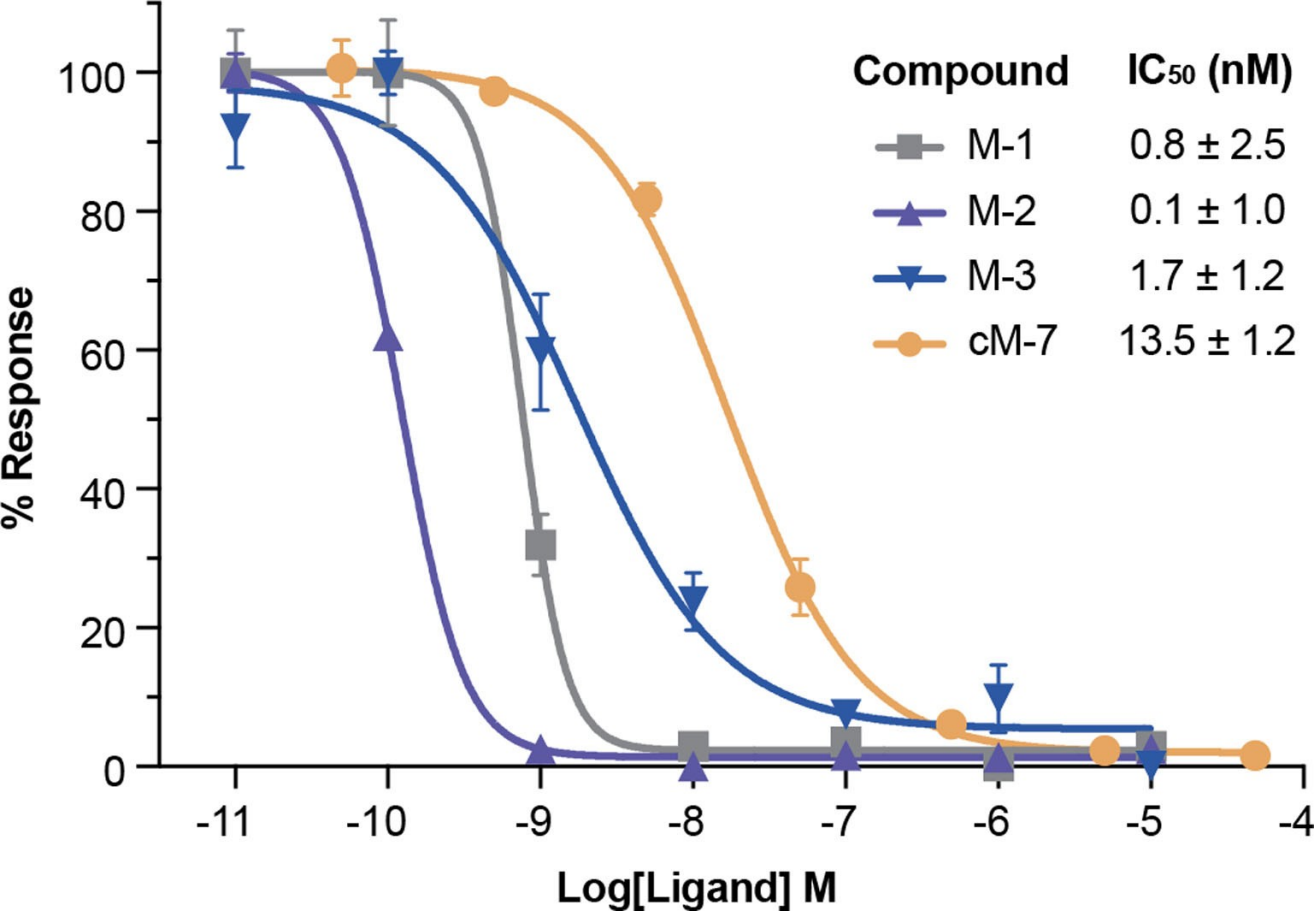
Activity assay of linear and cyclic MVIIA analogues against Ca_V_2.2 channels. Each data point was performed in triplicate and represents arithmetic means±standard error of the fit. The maximum tested concentration was 10 μM for M‐1, M‐2 and M‐3but was 50 μM for cM‐7.

Importantly, all of the cyclic analogues except cM‐9 were more active than their linear precursors (Figure S10), indicating that cyclization has a benefit when directly comparing linear vs cyclic molecules of similar amino acid composition, presumably deriving from entropic stabilization. In contrast, M‐9 displayed 10‐fold higher potency than cM‐9 (IC_50_=31.9±2.6 nM; *n*=7), highlighting that a longer linker length no longer engenders an entropic advantage in the peptide activity. Thus, through careful optimization of the linker and core peptide, we were able to generate potent cyclic analogues of MVIIA. The importance of the linker for activity and stability has also recently been demonstrated for other drug candidates, for example, proteolysis targeting chimeras (PROTACs).^[23]^

The stability of the native peptide M‐1 and cyclic analogue cM‐7 was evaluated in human serum and stimulated intestinal fluid (SIF). Both linear and cyclic MVIIA were highly resistant to degradation in serum, with more than 80 % and 95 % of peptide remaining respectively after 24 h (Figure 4A). The estimated half‐life of cyclic peptide cM‐7 was 41 hours, which was significantly higher than that of M‐1 (6 h), indicating that the stability of the native peptide can be substantially improved by backbone cyclization. In the SIF stability assay, a highly aggressive environment in that most peptides do not survive for long, cM‐7 had an estimated half‐life of 43 min with approximately 38 % remaining after 1 h. By contrast, the linear M‐1 degraded more rapidly, with a half‐life of only 9 min (Figure 4B). The superior metabolic stability of the cyclic analogue cM‐7 highlights the value of cyclization for improving the properties of therapeutic peptides.

**Figure 4:**
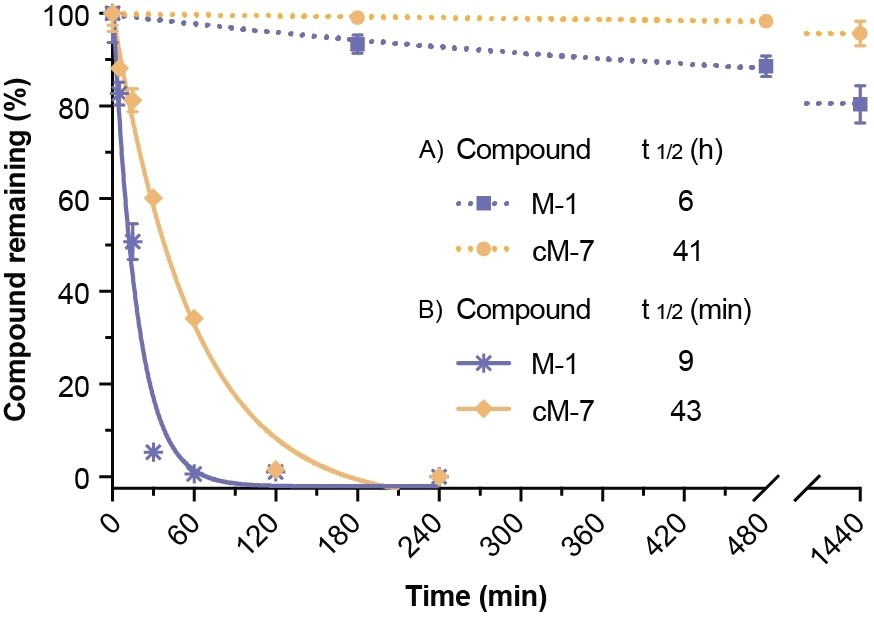
Metabolic degradation and calculated half‐lives (*t*_1/2_) of M‐1 and cM‐7 in A) human serum and B) stimulated intestinal fluid stability assay. Data were fitted to an exponential decay curve. Each data point represents the mean±standard deviation (*n*=3) of the normalized curve.

We note that in clinical practice ziconotide is delivered intrathecally via a surgically implanted slow‐release pump, in part to avoid potential side effects from interactions with peripheral targets, and that the intent of the current work was not to attempt to facilitate oral delivery to the systemic circulation followed by uptake through the blood brain barrier (BBB). Therefore, we have not examined the permeability or BBB penetration properties of the cyclized analogues. However, there is emerging evidence that Ca_V_2.2 inhibitors (such as MVIIA) can elicit peripherally mediated analgesia without the need for intrathecal administration or the need to cross the BBB.^[24]^ It is too early to speculate whether peripheral administration of MVIIA is a viable therapeutic route, in particular, because this effect may be pain‐type‐specific (for example, it may be limited to chemotherapy‐induced neuropathy).

In summary, we have developed a chemoenzymatic approach for efficiently producing cyclic MVIIA analogues which could not be obtained by chemical synthesis alone. The use of AEPs allows backbone cyclization to be carried out under mild conditions that generally do not interfere with already formed disulfides. The best analogue, cyclic analogue cM‐7, exhibits much higher stability than the native MVIIA in both human serum and SIF. We also show that these cyclic analogues can maintain inhibition of the Ca_V_2.2 channel, although they were less active than the native substrate. Overall, we find AEPs can facilitate the cyclization of peptides to improve stability and maintain activities, which cannot be obtained by chemical synthesis. The results highlight the excellent potential of AEPs for peptide engineering and provide insight for further improving the therapeutic value of peptides by backbone cyclization.

## Conflict of interest

The authors declare no conflict of interest.

## Supporting information

As a service to our authors and readers, this journal provides supporting information supplied by the authors. Such materials are peer reviewed and may be re‐organized for online delivery, but are not copy‐edited or typeset. Technical support issues arising from supporting information (other than missing files) should be addressed to the authors.

Supporting Information

## Data Availability

The data that support the findings of this study are available from the corresponding author upon reasonable request.
