# Supplementary material for: A Chemoenzymatic Approach To Produce a Cyclic Analogue of the Analgesic Drug MVIIA (Ziconotide)
Source: Angew Chem Int Ed Engl. 2023 May 31;62(29):e202302812. doi: 10.1002/anie.202302812 (PMC10952433; doi:10.1002/anie.202302812)
Supplement: Supplementary file 1 — Supporting Information [file ANIE-62-0-s001.pdf]

Angewandte Chemie
Eine Zeitschrift der Gesellschaft Deutscher Chemiker

Supporting Information

A Chemoenzymatic Approach To Produce a Cyclic Analogue of the Analgesic Drug MVIIA (Ziconotide)

Y. Zhou, P. J. Harvey, J. Koehbach, L. Y. Chan, A. Jones, Å. Andersson, I. Vetter, T. Durek, D. J. Craik**

Table of Contents

Section 1: Peptide synthesis 3
Section 2: Oxidation of ω-conotoxin MVIIA 5
Section 3: AEP-mediated cyclization 6
Section 4: Reduction alkylation 8
Section 5: NMR spectroscopy 9
Section 6: Oxidation of MVIIA 18
Section 7: FLIPR assay 19
Section 8: Serum stability assay 21
Section 9: Stimulated intestinal assay 22
Reference

Section 1: Peptide synthesis

Both the linear peptides (M-1, M-2 and M-3) and cyclic precursor peptides (M-5, M-6, M-7, M-8 and M-9) were synthesized using standard Fmoc solid-phase peptide synthesis (SPPS) on acid-labile 2-chlorotrityl chloride resin (2-CTC). Peptides were cleaved off the resin with 1% trifluoroacetic acid (TFA) in dichloromethane (DCM) (v/v) leaving the side chain protecting group intact. The side chain deprotection was conducted in TFA with 2.5% triisopropylsilane (TIPS) and 2.5% H_2O (v/v) before further purification by C18 reverse phase-high-performance liquid chromatography (RP-HPLC) and oxidation. The majority of TFA was evaporated under a vacuum, and the peptide precipitated with ice-cold diethyl ether. The peptide was dissolved in 50% acetonitrile with 0.05% TFA and lyophilized.

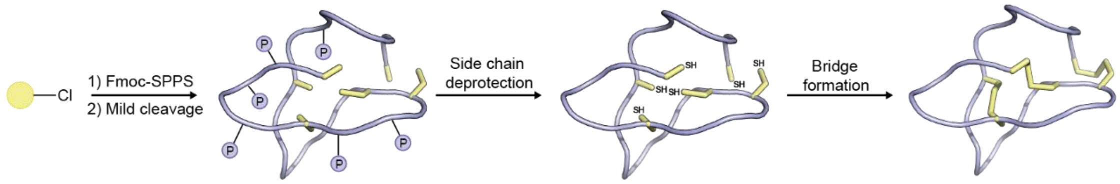

Figure S1. An overview of the synthetic strategy of MVIIA. All peptides were assembled using acid-labile 2-chlorotrityl chloride resin (yellow round circle). Side chain protecting groups (P) was removed subsequently generating the thioester for random oxidation.

Table S1. Peptide sequences and the monoisotopic mass.

Compound	Sequence	Mass[a] Calc. (Da) [M+H]$^+$	Obs. (Da)[b] [M+H]$^+$	Purity (%)[c]
M-1	H-CKGKGAKCSRLMYDCCTGSCRSGKC-NH$_2$	2639.1293	2638.6992	≥ 88
M-2	H-CKGKGAKCSRLNYDCCTGSCRSGKC-NH$_2$	2621.1293	2620.7185	≥ 98
M-3	H-CKGKGAKCSRLMYDCCTGSCRSGKC-OH	2640.1366	2639.7097	≥ 87
M-5	**GA**CKGKGAKCSRLNYDCCTGSCRSGKC**AGN**GL	3161.4022	3162.2517	≥ 99
cM-5	c[**GA**CKGKGAKCSRLNYDCCTGSCRSGKC**AGN**]	2973.2967	-	-
M-6	**GGA**CKGKGAKCSRLNYDCCTGSCRSGKC**AGN**GL	3218.4237	3219.2522	≥ 98
cM-6	c[**GGA**CKGKGAKCSRLNYDCCTGSCRSGKC**AGN**]	3030.3182	3030.1467	≥ 99
M-7	**GGAA**CKGKGAKCSRLNYDCCTGSCRSGKC**AGN**GL	3289.4535	3289.3447	≥ 98
cM-7	c[**GGAA**CKGKGAKCSRLNYDCCTGSCRSGKC**AGN**]	3101.3553	3102.0271	≥ 99
M-7m	**GGAA**CKGKGAKCSRLMYDCCTGSCRSGKC**AGN**GL	3306.4535	3306.7356	≥ 84
cM-7m	c[**GGAA**CKGKGAKCSRLMYDCCTGSCRSGKC**AGN**]	3118.3480	3118.6223	≥ 90
M-8	**GGAA**CKGKGAKCSRLNYDCCTGSCRSGKC**AGGN**GL	3346.4823	3347.2747	≥ 98
cM-8	c[**GGAA**CKGKGAKCSRLNYDCCTGSCRSGKC**AGGN**]	3158.3767	3159.1069	≥ 99
M-9	**GGAA**CKGKGAKCSRLNYDCCTGSCRSGKC**AAGGN**GL	3417.5194	3417.9067	≥ 96
cM-9	c[**GGAA**CKGKGAKCSRLNYDCCTGSCRSGKC**AAGGN**]	3229.4139	3229.1254	≥ 91

[a] Based on the monoisotopic mass. [b] Observed by MALDI-TOF-MS. [c] Determined by RP-HPLC using a gradient from 0-60% solvent B in solvent A over 35 min (Solvent A: 0.05% TFA in H_2O; Solvent B: 0.05% TFA, 10% H_2O, 90% acetonitrile).

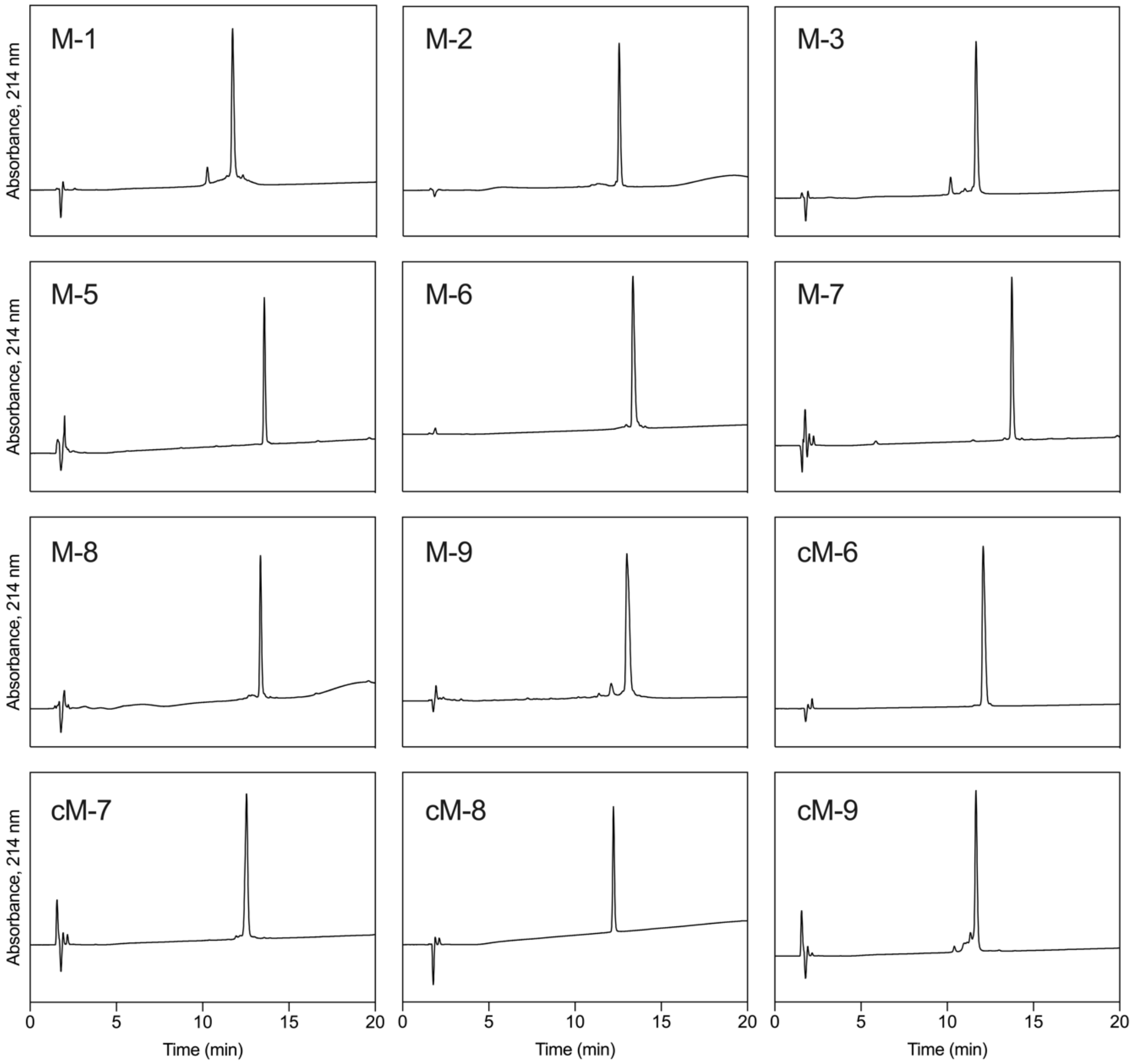

Figure S2. Analytical LC Chromatograms. Conditions: Gradient of 5-45% solvent B (0.05% TFA, 10% H_2O, 90% acetonitrile) in solvent A (0.05% TFA in H_2O) over 35 min (C18 column, 5 μm, 300 Å, 150 × 2.1mm).

Section 2: Oxidation of ω-conotoxin MVIIA

The purified linear and cyclic analogues were oxidized in Buffer 1 (0.33 M NH_4OAc, 0.5 M GnHCl; 50 mM GSH; 5 mM GSSG; pH 6.5) with a final peptide concentration of 1 mg/mL or Buffer 2 (0.1 M NH_4HCO_3; pH 8.5) with a final concentration of 0.1 mg/mL. Folding reactions were conducted at 4 °C for 2 days. RP-HPLC was used to purify the peptides, and the molecular masses were confirmed by ESI-MS.

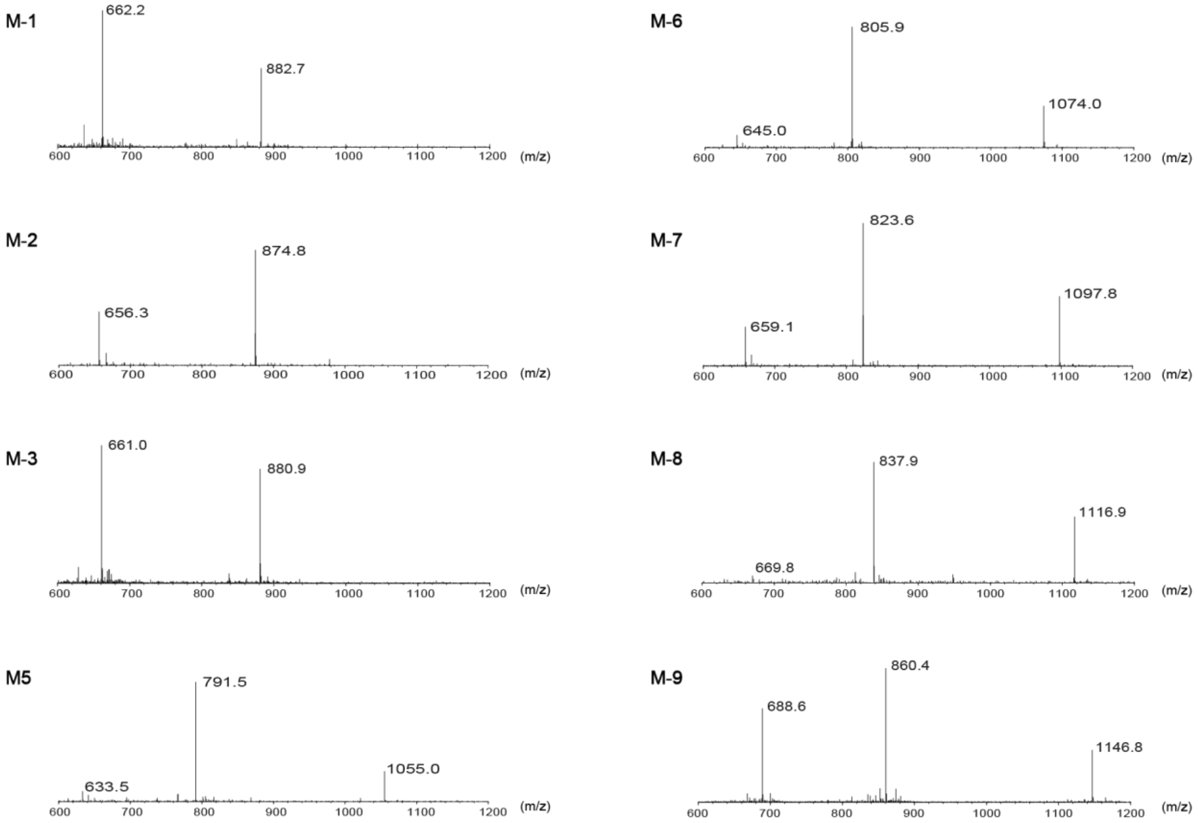

Figure S3. ESI-MS mass spectrum of linear peptides M-1, M-2 and M-3, and cyclic precursor peptides M-5, M-6, M-7, M-8 and M-9 after RP-HPLC purification.

Section 3: AEP-mediated cyclization

The properly folded cyclic precursor peptides (100 μM) were incubated with *Oa*AEP1 (1 μM) at pH 6.5 at room temperature for 48 hours, followed by analytical RP-HPLC for purification. Reactions were run in 0.1 M sodium phosphate buffer with 0.1 M sodium chloride and 1 mM ethylenediaminetetraacetic acid (EDTA). Before purification, 1 μL of each reaction was taken and quenched into 9 μL of 2% TFA. The mixtures were injected and analyzed by AB Sciex 5600 TripleTOF MS.

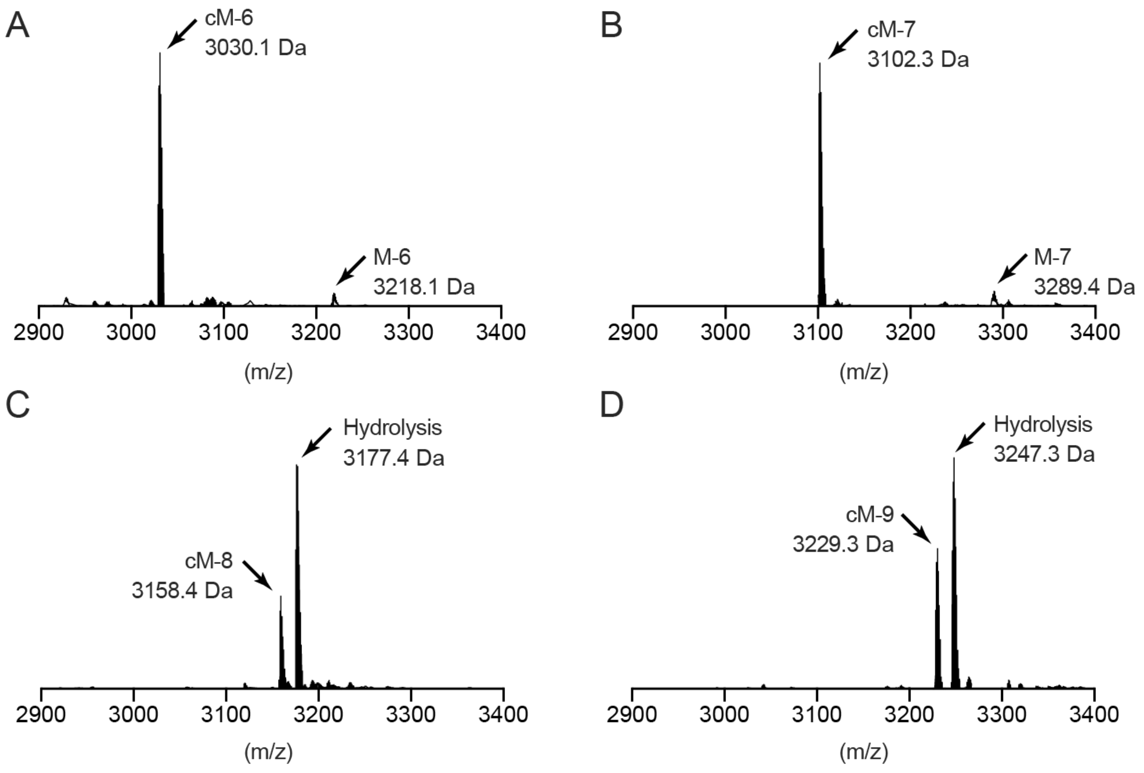

Figure S4. AB Sciex 5600 TripleTOF MS profile of cyclization reaction. Mass reconstructed spectra were shown. A) M-6 $[M + H]^+$ observed = 3218.1 Da; cM-6 $[M + H]^+$ observed = 3030.1 Da. B) M-7 $[M + H]^+$ observed = 3289.4 Da; cM-7 $[M + H]^+$ observed = 3102.3 Da. C) M-8 $[M + H]^+$ observed = 3177.4 Da; cM-8 $[M + H]^+$ observed = 3158.4 Da. D) M-9 $[M + H]^+$ observed = 3247.3 Da; cM-9 $[M + H]^+$ observed = 3229.3 Da.

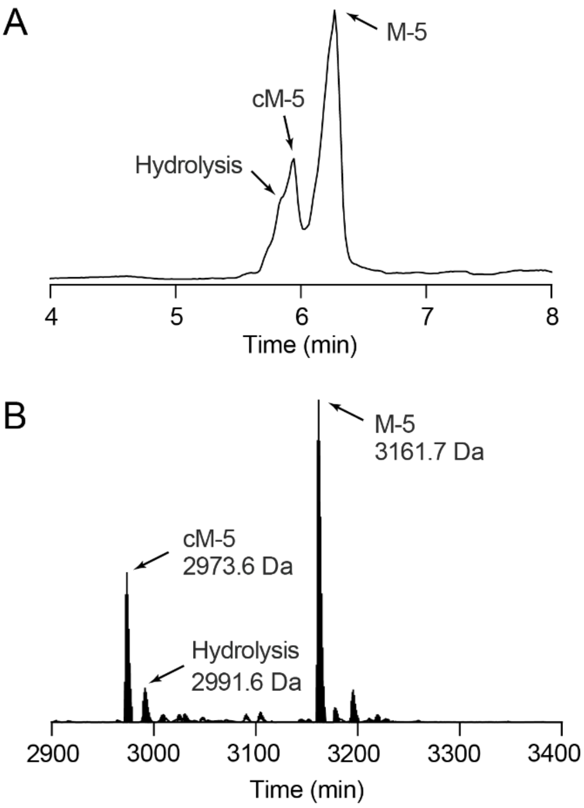

Figure S5. AEP-mediated cyclization of M-5. A) AB Sciex 5600 TripleTOF MS profile of cyclization reaction. B) Mass reconstructed spectra were shown. M-5 $[M + H]^+$ observed = 3161.7 Da; cM-5 $[M + H]^+$ observed = 2973.6 Da; hydrolysis $[M + H]^+$ observed = 2991.6 Da.

Section 4: Reduction alkylation

The cyclic analogue cM-7 was redissolved in 100 µL ammonium bicarbonate (pH 8.5) to give a final peptide concentration of 10-20 µM. 100 mM of DTT was added and the mixtures were incubated at 60 °C for 30 min to reduce the peptides. After cooling down to room temperate, 25 mM of iodoacetamide was added and the mixtures were kept in the dark at room temperature for 30 min for alkylation. 50 µL of the mixture was removed to another tube and 5 µL trypsin was added to one portion and incubated at 37 °C overnight. The mixtures were injected and analyzed by AB Sciex 5600 TripleTOF MS. Peptide fragments obtained from the reduction, alkylation and trypsin-digestion of cyclic peptides were fragmented using MS/MS and the spectra were manually assigned.

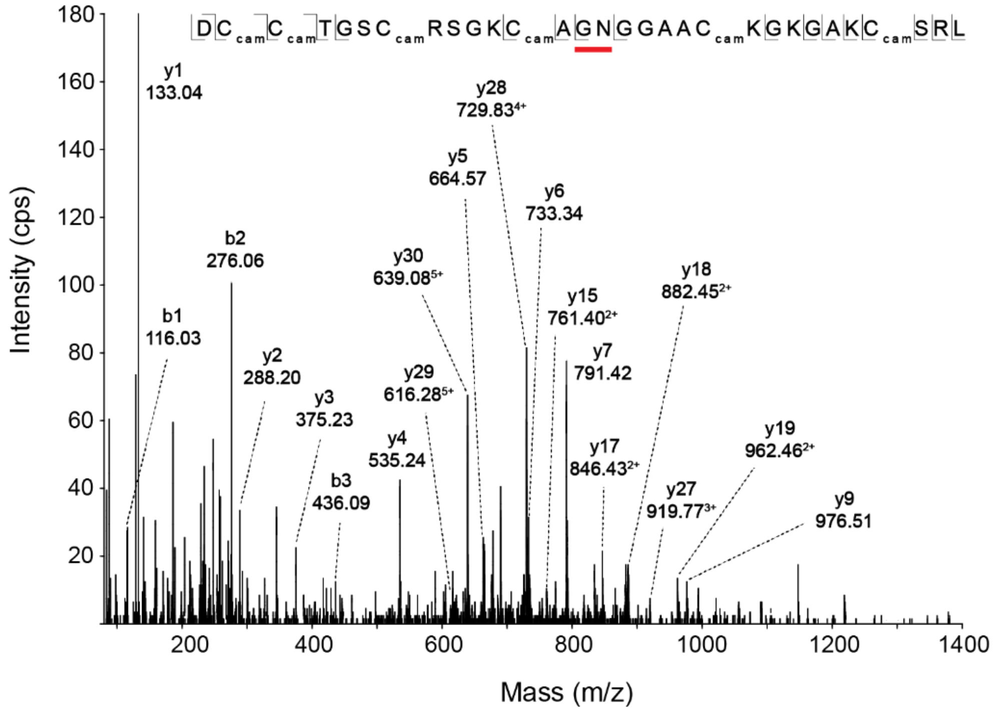

Figure S6. Peptide mass fingerprinting of cM-7. Peptide fragments were subjected to MS/MS fragmentation after reduction, alkylation and chymotrypsin-digestion of cyclic peptides and the resulting spectra were assigned to confirm cyclization.

Section 5: NMR spectroscopy

1D and 2D NMR spectroscopy were conducted on a Bruker AVANCE III HD 600 MHz spectrometer equipped with a cryogenically cooled probe. Lyophilized peptides were prepared in 90% H_2O and 10% D_2O (v/v) and experiments were run at 298 K. Chemical shifts were referenced to internal 2,2-dimethyl-2-silapentane-5-sulfonate at 0 ppm. 1D proton spectra were obtained for each peptide sample. For cM-7, additional 2D TOCSY, NOESY, ^{15}N and ^{13}C HSQC spectra were obtained and the spectra were manually assigned using CCPNMR analysis 2.4.2. Variable temperature coefficient experiments were performed in H_2O/D_2O (9:1) with ^1H and TOCSY experiments run at a series of temperatures (283, 288, 293, 298 and 303 K). The amide resonance for each residue was plotted against temperature and temperature coefficient values greater than -4.7 ppb/K were indicative of solvent protection.[1]

D_2O exchange experiments were conducted by preparing the sample in 100% D_2O and monitoring the exchange of amide protons by ^1H and TCOSY experiments over 24 h. The presence of an amide resonance for greater than 2 hours was defined as slow exchange and indicative of H-bonding.

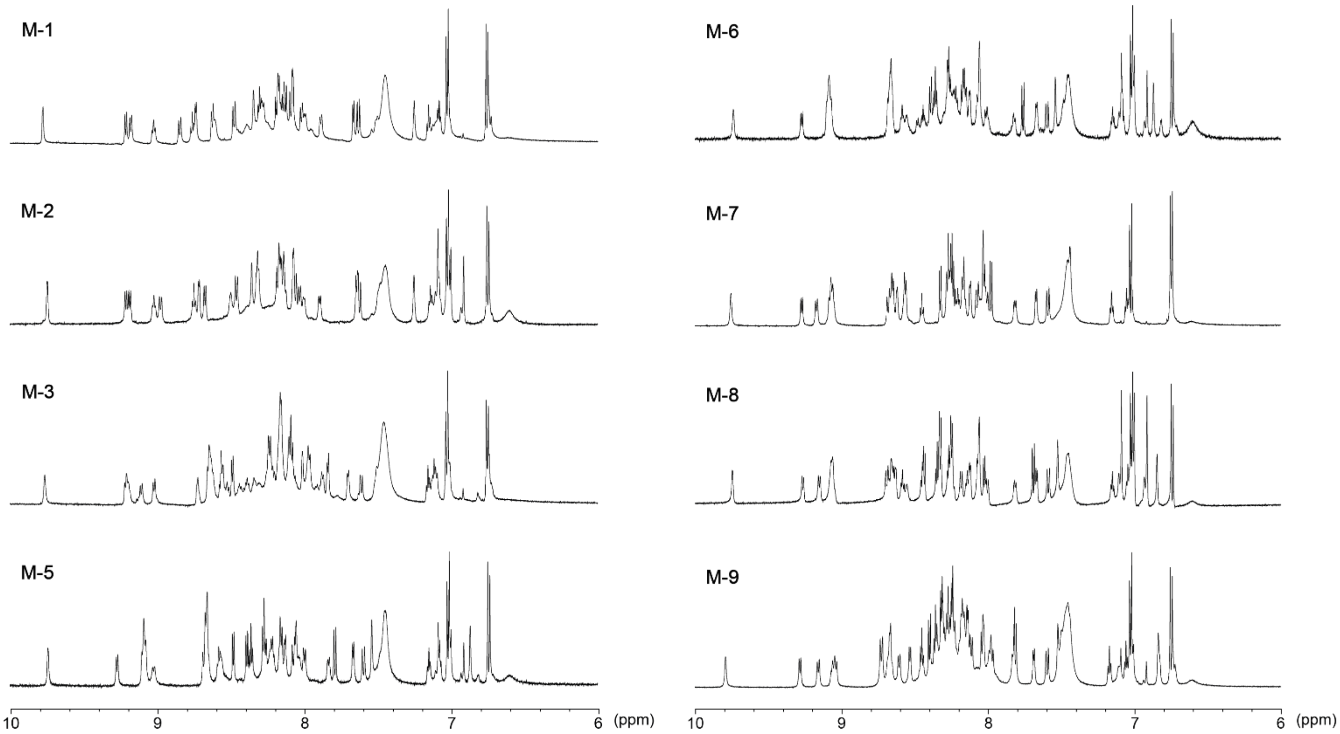

Figure S7. 1D ^{1}H-NMR spectrum of folded MVIIA analogues. All the spectrums were well-dispersed on the amide chemical shift regions.

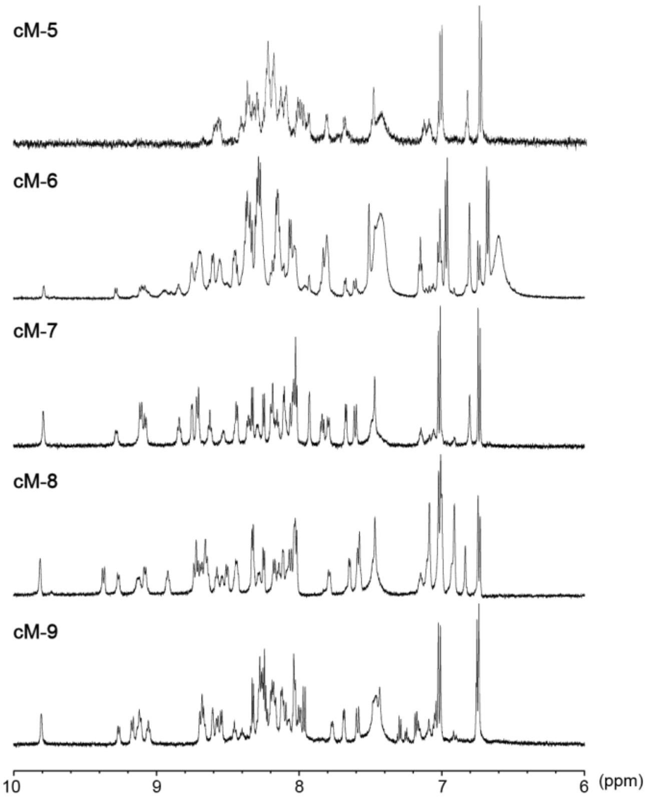

Figure S8. 1D ^{1}H-NMR spectrum of cyclic MVIIA analogues. All the spectrums except cM-5 were well-dispersed which confirmed the properly folded structures of the corresponding peptide species.

Table S2. Comparison of shifts between cM-7 and native MVIIA from literature

		cM-7 (25 °C, pH 3.5)				Kohno *et al*, Biochem 1995 (15 °C, pH 3.5)	Atkinson *et al*, Biochem 2000 (10 °C, pH 3.5)
	Residue	Shift (ppm)	Atom		Residue	Shift (ppm)	Shift (ppm)
1	G	120.748	N				
1		7.927	HN				
1		40.926	CA				
1		3.277	HA2				
1		3.142	HA3				
2	G	108.689	N				
2		8.541	HN				
2		44.819	CA				
2		4.015	HA2				
2		4.015	HA3				
3	A	124.155	N				
3		8.338	HN				
3		52.227	CA				
3		4.34	HA				
3		1.395	QB				
3		19.398	CB				
4	A	124.078	N				
4		8.415	HN				
4		52.003	CA				
4		4.408	HA				
4		1.398	QB				
4		19.065	CB				
5	C	8.115	HN	1	C		
5		54.331	CA	1			52.62
5		4.878	HA	1		4.61	4.63
5		42.533	CB	1			39.7
5		3.142	HB2	1		3.28	3.26
5		2.995	HB3	1		3.1	3.11
6	K	120.595	N	2	K		
6		9.151	HN	2		9.33	9.42
6		55.281	CA	2			54.19
6		4.416	HA	2		4.49	4.48
6		34.575	CB	2			33.89
6		1.837	HB2	2		1.89	1.88
6		1.837	HB3	2		1.83	1.8
6		25.099	CG	2			
6		1.561	HG2	2		1.5	1.48
6		1.415	HG3	2		1.37	1.34
6		28.27	CD	2			27.03
6		1.644	HD2	2		1.67	1.65
6		1.564	HD3	2		1.58	1.58

7	G	111.149	N	3	G		
7		8.718	HN	3		8.94	9
7		43.834	CA	3			42.28
7		4.087	HA2	3		4.04	4.04
7		3.769	HA3	3		3.79	3.8
8	K	118.623	N	4	K		
8		8.018	HN	4		8.23	8.3
8		58.258	CA	4			56.8
8		3.726	HA	4		3.78	3.78
8		32.865	CB	4			30.55
8		1.644	HB2	4		1.73	1.75
8		1.605	HB3	4		1.7	1.67
8		1.242	HG2	4		1.34	1.49
8		1.242	HG3	4		1.34	1.32
8		31.243	CD	4			
8		1.519	HD2	4		1.5	1.67
8		1.471	HD3	4		1.5	1.67
9	G	114.211	N	5	G		
9		8.944	HN	5		9.23	9.27
9		45.534	CA	5			43.5
9		4.216	HA2	5		4.35	4.32
9		3.648	HA3	5		3.56	3.56
10	A	123.054	N	6	A		
10		7.77	HN	6		7.87	7.82
10		51.671	CA	6			50.18
10		4.272	HA	6		4.28	4.27
10		1.414	QB	6		1.22	1.42
10		20.823	CB	6			18.85
11	K	119.484	N	7	K		
11		8.203	HN	7		8.34	8.38
11		56.964	CA	7			55.31
11		4.681	HA	7		4.67	4.69
11		32.57	CB	7			30.99
11		1.75	HB2	7		1.77	1.77
11		1.75	HB3	7		1.77	1.77
11		1.42	HG2	7		1.46	1.65
11		1.42	HG3	7		1.46	1.42
11		1.64	HD2	7		1.65	1.72
11		1.64	HD3	7		1.65	1.72
12	C	118.493	N	8	C		
12		8.153	HN	8		8.23	8.25
12		53.267	CA	8			51.5
12		4.992	HA	8		5.02	5.03
12		45.883	CB	8			43.67
12		3.312	HB2	8		3.28	3.29
12		2.923	HB3	8		2.92	2.88

13	S	115.514	N	9	S		
13		9.043	HN	9		8.93	9.19
13		56.959	CA	9			54.5
13		4.656	HA	9		4.72	4.71
13		64.824	CB	9			63.07
13		3.87	HB2	9		3.88	3.84
13		3.739	HB3	9		3.74	3.73
14	R	124.771	N	10	R		
14		8.656	HN	10		8.9	8.99
14		57.506	CA	10			56.94
14		4.165	HA	10		4.02	4
14		30.587	CB	10			28.57
14		1.848	HB2	10		1.87	1.85
14		1.848	HB3	10		1.87	1.85
14		27.077	CG	10			25.43
14		1.642	HG2	10		1.62	1.59
14		1.642	HG3	10		1.62	1.59
14		43.307	CD	10			43.61
14		3.244	HD2	10		3.26	
14		3.243	HD3	10		3.26	
14		7.243	HE	10			
15	L	8.142	HN	11	L	8.12	8.19
15		56.385	CA	11			54.19
15		4.117	HA	11		4.07	4.01
15		41.893	CB	11			39.7
15		1.613	HB2	11		1.61	1.62
15		1.614	HB3	11		1.61	1.61
15		-	HG	11		1.61	1.55
15		0.923	QD1	11		0.91	0.92
15		0.888	QD2	11		0.91	0.88
15		24.779	CD1	11			23.28
15		23.225	CD2	11			21.19
16	norL	118.725	N	12	M		
16		7.54	HN	12		7.51	7.55
16		56.013	CA	12			53.85
16		4.278	HA	12		4.44	4.42
16		33.88	CB	12			31.17
16		1.51	HB2	12		1.92	1.91
16		1.51	HB3	12		1.92	1.91
16		23.9	CG	12			30.65
16		1.2	HG2	12			2.48
16		1.2	HG3	12			2.32
16		29.83	CD				
16		1.16	HD2				
16		1.03	HD3				
16		15.8	CE				

16		0.8	QE				
17	Y	117.133	N	13	Y		
17		7.892	HN	13		7.92	7.91
17		58.301	CA	13			57.08
17		4.51	HA	13		4.5	4.46
17		36.742	CB	13			34.66
17		3.214	HB2	13		3.25	3.27
17		2.997	HB3	13		3.01	2.98
17		7.11	QD	13		7.11	7.1
17		6.833	QE	13		6.94	3.83
18	D	8.117	HN	14	D	8.15	8.12
18		54.524	CA	14			52.12
18		4.742	HA	14		4.81	1.79
18		41.807	CB	14			39.28
18		2.926	HB2	14		2.97	2.99
18		2.583	HB3	14		2.53	2.47
19	C	118.594	N	15	C		
19		8.349	HN	15		8.45	8.58
19		55.454	CA	15			54.02
19		4.924	HA	15		4.91	4.88
19		38.781	CB	15			35.83
19		3.172	HB2	15		3.14	3.1
19		2.578	HB3	15		2.66	2.65
20	C	125.009	N	16	C		
20		9.884	HN	16		9.97	9.97
20		57.376	CA	16			55.65
20		4.372	HA	16		4.4	4.4
20		39.592	CB	16			37.65
20		3.226	HB2	16		3.24	3.25
20		2.904	HB3	16		2.93	2.91
21	T	109.565	N	17	T		
21		8.287	HN	17		8.47	8.39
21		60.426	CA	17			59.07
21		4.482	HA	17		4.49	4.5
21		70.781	CB	17			69.21
21		4.188	HB	17		4.1	4.06
21		1.127	QG2	17		1.13	1.13
21		21.263	CG2	17			19.32
22	G	108.805	N	18	G		
22		8.238	HN	18		8.45	8.55
22		45.386	CA	18			43.67
22		4.254	HA2	18		4.12	4.13
22		3.823	HA3	18		3.81	3.8
23	S	119.02	N	18			
23		8.12	HN	19	S	8.3	8.38
23		56.902	CA	19			55.23

23		4.719	HA	19		4.74	4.75
23		65.389	CB	19			63.94
23		3.775	HB2	19		3.81	3.8
23		3.684	HB3	19		3.72	3.71
24	C	123.47	N	20	C		
24		8.798	HN	20		8.75	8.75
24		55.676	CA	20			54.25
24		4.808	HA	20		4.72	4.69
24		40.634	CB	20			39.08
24		3.026	HB2	20		3.02	3.07
24		2.869	HB3	20		2.86	2.83
25	R	131.15	N	21	R		
25		8.813	HN	21		8.61	8.62
25		55.072	CA	21			53.66
25		4.677	HA	21		4.67	4.68
25		30.545	CB	21			30.13
25		1.85	HB2	21		1.87	1.88
25		1.657	HB3	21		1.81	1.8
25		26.79	CG	21			25.29
25		1.65	HG2	21		1.65	1.65
25		1.52	HG3	21		1.55	1.55
25		43.288	CD	21			41.63
25		3.16	HD2	21		3.2	3.22
25		3.16	HD3	21		3.2	
25		7.152	HE	21			
26	S	121.505	N	22	S		
26		9.375	HN	22		9.38	9.45
26		58.404	CA	22			56.8
26		4.025	HA	22		4.06	4.06
26		61.783	CB	22			60.08
26		4.141	HB2	22		4.13	4.13
26		3.88	HB3	22		3.92	3.91
27	G	103.902	N	23	G		
27		8.276	HN	23		8.35	8.43
27		45.647	CA	23			44.01
27		4.138	HA2	23		4.14	4.14
27		3.782	HA3	23		3.82	3.8
28	K	120.583	N	24	K		
28		7.709	HN	24		7.73	7.77
28		54.92	CA	24			53.41
28		5.302	HA	24		5.33	5.34
28		37.173	CB	24			35.46
28		1.563	HB2	24		1.6	1.59
28		1.493	HB3	24		1.51	1.48
28		25.03	CG	24			23.38
28		1.34	HG2	24		1.37	1.36

28		1.25	HG3	24		1.27	1.25
28		29.08	CD	24			27.77
28		1.68	HD2	24		1.7	1.69
28		1.68	HD3	24		1.7	
28		2.992	HE2	24		3.01	
28		2.992	HE3	24		3.01	
29	C	121.592	N	25	C		
29		8.857	HN	25		8.84	8.82
29		55.598	CA	25			53.18
29		4.833	HA	25		4.82	4.82
29		37.497	CB	25			35.83
29		3.25	HB2	25		3.23	3.24
29		3.086	HB3	25		3.09	3.09
30	A	131.033	N				
30		9.197	HN				
30		51.681	CA				
30		4.653	HA				
30		1.498	QB				
30		20.591	CB				
31	G	109.449	N				
31		8.441	HN				
31		45.508	CA				
31		4.197	HA2				
31		3.801	HA3				
32	N	119.301	N				
32		8.527	HN				
32		54.08	CA				
32		4.458	HA				
32		38.199	CB				
32		2.804	HB2				
32		2.707	HB3				
32		112.563	ND2				
32		7.561	HD21				
32		6.898	HD22				

Table S3. Variable temperature coefficient and D_2O exchange data (chemical shifts in ppm).

	Residue	283	288	293	298	303	Temperature coefficient (ppb/K)
1	G	7.975	7.929	7.888	7.842	7.808	-8.42
2	G	8.542	8.512	8.486	8.456	8.432	-5.52
3	A	8.37	8.332	8.295	8.255	8.225	-7.34
4	A	8.446	8.408	8.372	8.334	8.303	-7.20
5	C	8.159	8.113	8.075	8.025	7.996	-8.28
6	K	9.176	9.138	9.104	9.083	9.042	-6.46
7	G	8.746	8.708	8.674	8.632	8.6	-7.36
8	K			7.971	7.932	7.903	-6.80
9	G	8.921	8.898	8.882	8.851	8.84	-4.18
10	A	7.738	7.72	7.704	7.681	7.672	-3.42[a]
11	K	8.222	8.182	8.152	8.113	8.094	-6.50
12	C	8.122	8.108	8.089	8.061	8.058	-3.50[a]
13	S	9.133	9.093	9.05		8.966	-8.38
14	R			8.6	8.536	8.517	-8.30
15	L	8.114	8.103	8.087		8.066	-2.43
16	norL				7.495	7.471	-4.80
17	Y	7.855	7.84	7.832	7.801	7.796	-3.14
18	D	8.052	8.049	8.047	8.044	8.029	-1.02
19	C		8.329	8.308	8.297	8.249	-5.02
20	C			9.826	9.804	9.782	-4.40
21	T		8.259	8.231	8.203	8.174	-5.66
22	G		8.241	8.2	8.16	8.124	-7.82
23	S	8.13	8.099	8.07	8.037	8.011	-6.00
24	C		8.774	8.746	8.716	8.689	-5.70
25	R	8.774	8.761	8.748	8.722	8.717	-3.06
26	S		9.367	9.331	9.286	9.253	-7.74
27	G		8.282	8.238	8.187	8.153	-8.76
28	K	7.651	7.638	7.63	7.612	7.607	-2.28[a]
29	C	8.777	8.777	8.777	8.762	8.772	-0.50[a]
30	A		9.178	9.147	9.116	9.087	-6.08
31	G		8.432	8.397	8.361	8.326	-7.08
32	N			8.482	8.443	8.415	-6.70

[a] Slow D_2O exchange.

Section 6: Oxidation of cyclic MVIIA

The cyclic MVIIA (30 µM) used for folding trials had the same 7-residue linker as cM-7. After chemical synthesis and side-chain deprotection, six folding conditions were tested. Condition 1: 75% (v/v) 0.33 M NH_4OAc, 25% (v/v) isopropanol; pH 6.5. Condition 2: 0.33 M NH_4OAc, 0.5 M GnHCl, 50 mM GSH, 5 mM GSSG; pH 6.5. Condition 3: 0.33 M NH_4OAc, 0.5 M GnHCl, 50 mM GSH, 5 mM GSSG; pH 6.5; 90 µM peptide. Condition 4: 0.33 M NH_4OAc; pH 6.5. Condition 5: 0.1 M NH_4HCO_3; pH 8.5. Condition 6: 0.1 M NH_4HCO_3, 0.5 M GnHCl, 50 mM GSH, 5 mM GSSG; pH 8.5. cM-7 was treated as the positive control and the chemically synthesized cyclic MVIIA was used as the negative control. The difference in retention time between the controls and the folding trials was checked by AB Sciex 5600 TripleTOF MS and the ion extract was set between 3101 to 3103 Da.

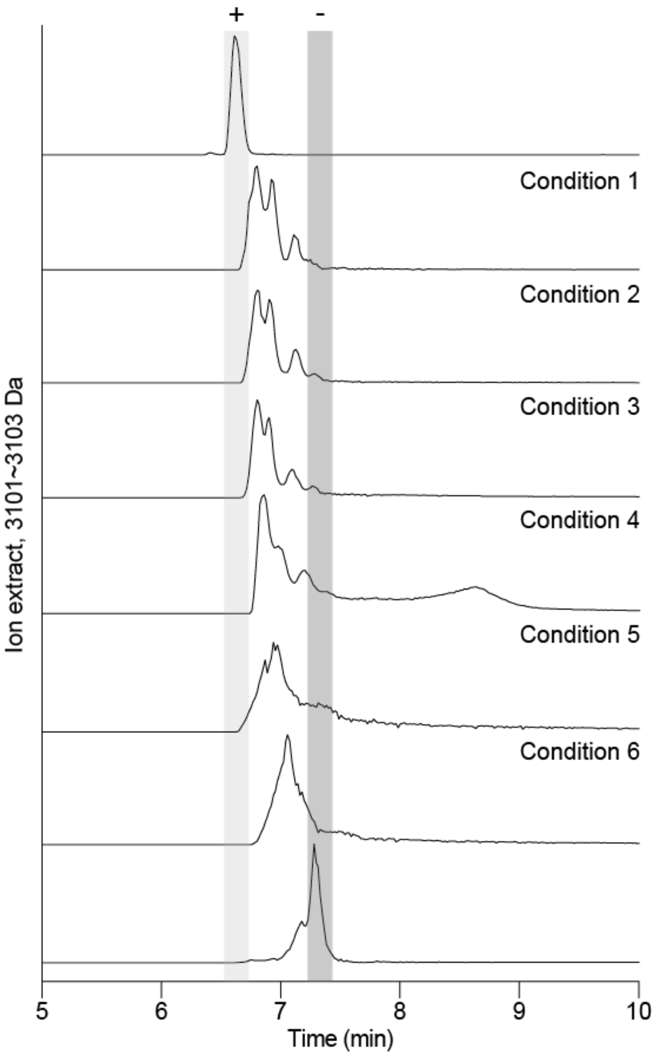

Figure S9. AB Sciex 5600 TripleTOF MS profiles of folding trials of cyclic MVIIA under six conditions. The positive control cM-7 had the shortest retention time (light grey) whereas the chemically synthesized cyclic MVIIA as the negative control had the longest retention (grey). The retention times of each folding trial were between the time of the positive control and the negative control.

Section 7: FLIPR assay

SH-SY5Y neuroblastoma cells were plated on a 384-well black wall clear bottom imaging plate at a density of 50,000 cells per well and incubated overnight at 37 °C in the presence of 5% carbon dioxide (CO_2). The media were flicked off from the wells and replaced with 20 µL of the loading dye. The loading dye was comprised of 5 µM of Calcium-4 No-wash dye (Molecular Devices, Sunnyvale, CA) and 10 µM of nifedipine in physiological salt solution (PSS) containing 5.9 mM KCl, 1.8 mM $CaCl_2$ and 0.1% bovine serum albumin (BSA) at pH 7.4. The cells were incubated for 30 min (37 °C; 5% CO_2) before the plate was transferred to the FLIPR^{TETRA} (Molecular Devices) to record the fluorescence responses. PSS (0.1% BSA) was used as negative control and the concentrations of the tested compounds ranged from 50 to 0.1 µM for the cyclic MVIIA analogues (10 to 0.01 µM for the linear MVIIA compounds). $Ca_V2.2$-mediated responses were elicited following a 5 min peptide incubation by the addition of PSS containing 5.9 mM KCl, 1.8 mM $CaCl_2$.

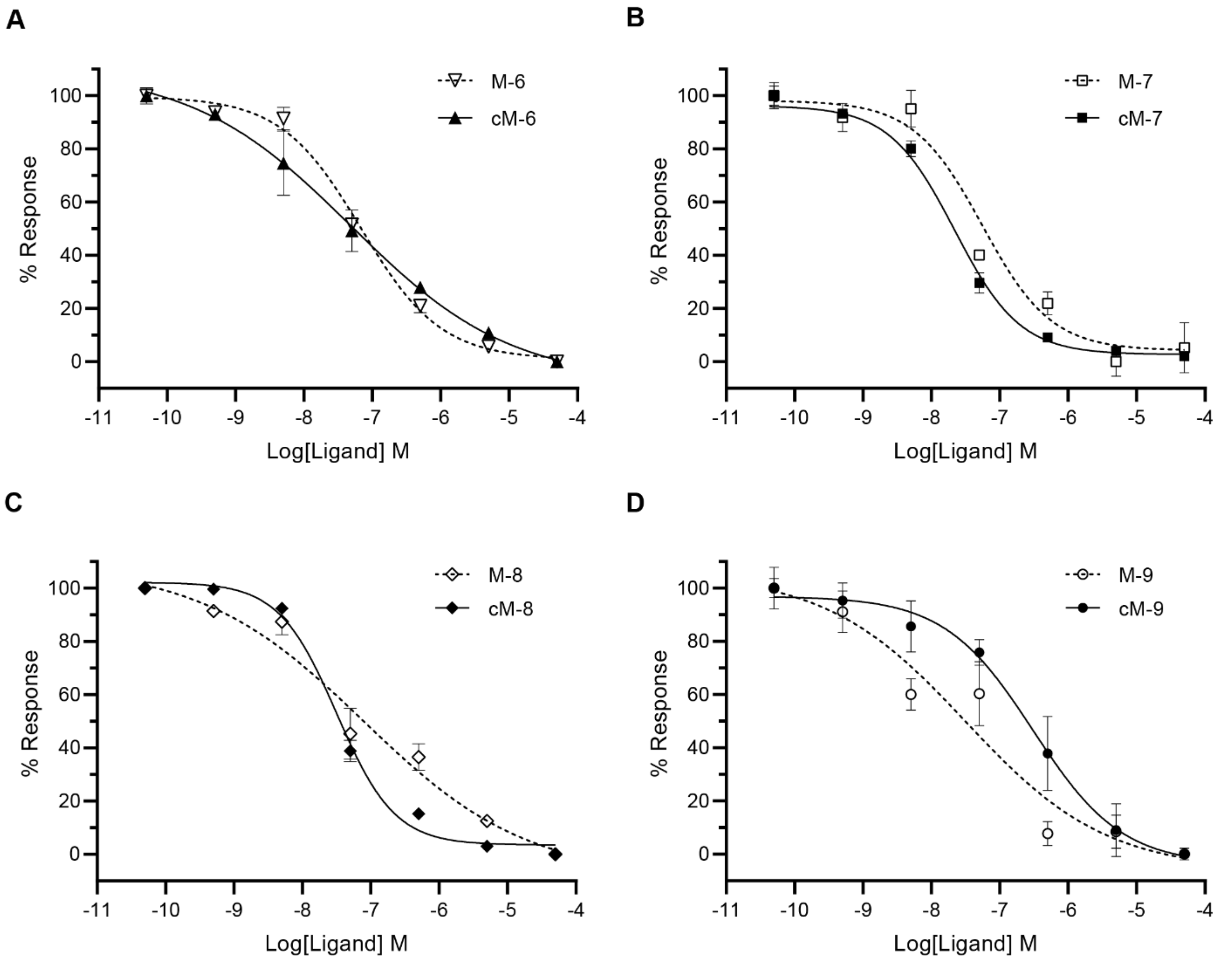

Figure S10. Linear and cyclic MVIIA inhibition of calcium channel. A) Concentration-response curves for M-6 and cM-6. B) Concentration-response curves for M-7 and cM-7. C) Concentration-response curve for M-8 and cM-8. D) Concentration-response curve for M-9 and c-9. The assay was done in one experiment with multiple wells. Error bars represent the mean ± SD.

Table S4. Comparison of EC_{50} (nM) determined for inhibition of the N-type Cav2.2 channels by linear and cyclic MVIIA analogues. Confidence intervals (95%) are indicated and mean ± SD for N-type Cav2.2 channels.

Compound	LogEC_{50}	EC_{50} (nM)
M-1	-9.1 ± 0.4	0.8 ± 2.5
M-2	-9.9 ± 0.02	0.1 ± 1.0
M-3	-8.8 ± 0.1	1.7 ± 1.2
M-6	-7.2 ± 0.1	67.8 ± 1.2
M-7	-7.3 ± 0.2	55.5 ± 1.7
M-8	-7.1 ± 0.2	78.7 ± 1.6
M-9	-7.5 ± 0.4	31.9 ± 2.6
cM-6	-7.4 ± 0.2	43.2 ± 1.4
cM-7	-7.8 ± 0.1	13.5 ± 1.2
cM-8	-7.6 ± 0.1	21.8 ± 1.2
cM-9	-6.4 ± 0.2	306.6 ± 1.5

Section 8: Human serum stability assay

Pooled human serum (Sigma-Aldrich, H4522, serum from male AB human plasma) was centrifuged at 13,000 rpm for 10 min to remove the lipid component. The supernatant was incubated for 15 min at 37 °C prior to the assay. Each peptide (200 μM) was diluted 1 in 10 in 100% human serum in triplicate and incubated at 37 °C. Test peptides were also diluted 1 in 10 in PBS and incubated in parallel for each time point as a control. 25 μL of aliquots were taken at 0, 3, 8, and 24 hours. To each serum aliquot, 50 μL of quench buffer (3% TFA in ACN) was added, and the samples were incubated for 10 min at 4 °C to precipitate serum proteins. The samples were centrifuged at 13,000 rpm for 5 min, and ~70 μL of the supernatant was taken. 500 μL of H_2O was added to the supernatant before the samples were analyzed by LC-MS/MS (AB Sciex 5600 TripleTOF MS). The elution time for each peptide was determined by the PBS control for that time point. The stability at each time point was calculated as the amplitude/area of the serum-treated peptide peak on LC-MS/MS as a percentage of the amplitude/area of the PBS-treated control peptides. The data were analyzed by GraphPad Prism using an "one phase decay" mode.

Section 9: Stimulated intestinal fluid stability assay

The stimulated intestinal fluid (SIF) composition met test solution criteria specified by the USP (USP 42 – NF 37, 2019). Preparation of SIF fluid, pH 6.8: KH_2PO_4 (6.8 mg/mL) was dissolved into 10 mL of ddH_2O, and the final pH (± 0.1) was adjusted with aq. NaOH (5 M). Pancreatin (100 mg, USP activity) was added, and the mixture was vortexed for 5 min and sonicated for 15 min at 25 °C. The suspension was centrifuged and syringe-filtered (0.45 µm) before use. The SIF fluid was incubated in a shaker at 37 °C for 15 min prior to the assay. Each peptide (200 µM) was diluted 1 in 10 in the SIF fluid in triplicate and incubated at 37 °C. Test peptides were also diluted 1 in 10 in PBS and incubated in parallel for each time point as a control. 25 µL of aliquots were taken at 0, 5, 15, 30, 60, 120 and 240 min. 50 µL of quench buffer (3% TFA in ACN) was added at each timepoint. The samples were centrifuged at 13,000 rpm for 5 min, and ~70 µL of the supernatant was taken. 500 µL of H_2O was added to the supernatant before the samples were analyzed by LC-MS/MS (AB Sciex 5600 TripleTOF MS). The elution time for each peptide was determined by the PBS control for that time point. The stability at each time point was calculated as the amplitude/area of the serum-treated peptide peak on LC-MS/MS as a percentage of the amplitude/area of the PBS-treated control peptides. The data was analyzed by GraphPad Prism using an “one phase decay” mode.

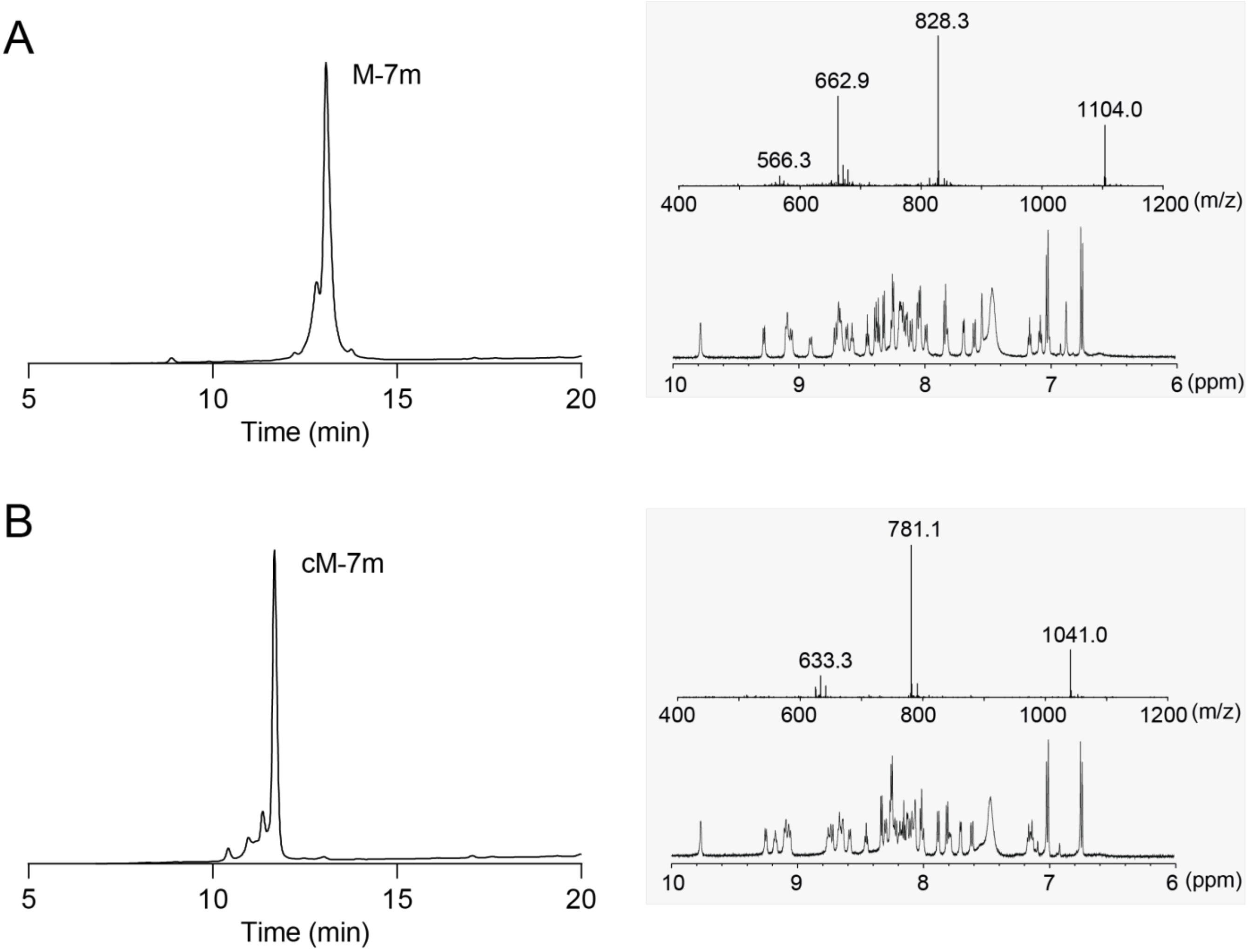

Figure S11. A chemo-enzymatic approach to produce cyclic M-7m: c[**GGAA**CKGKGAKCSRLMYDCCTGSCRSGKC**AGN]**. A) Oxidation of M-7m. The mass was shown by ESI-MS mass spectrometry ([M + H]$^{3+}$ calculated = 1103.2 Da; [M + H]$^{3+}$ observed = 1104.0 Da). 1D ^{1}H-NMR spectrum was well-dispersed which confirmed the correctly folded structure. B) Cyclization of M-7m. The mass was shown by ESI-MS mass spectrometry ([M + H]$^{3+}$ calculated = 1040.5 Da; [M + H]$^{3+}$ observed = 1041.0 Da). 1D ^{1}H-NMR spectrum of cM-7m was well-dispersed on the amide chemical shift regions.

References

[1] N. J. Baxter, M. P. Williamson, *J. Biomol. NMR* **1997**, *9*, 359-369.
[2] T. Kohno, J. I. Kim, K. Kobayashi, Y. Kodera, T. Maeda, K. Sato, *Biochemistry* **1995**, *34*, 10256-10265.
[3] R. A. Atkinson, B. Kieffer, A. Dejaegere, F. Sirockin, J.-F. Lefèvre, *Biochemistry* **2000**, *39*, 3908-3919.
